# The direct effects of media exposure on behaviors aimed at preventing COVID-19 and its indirect effects as mediated by interpersonal communication: a longitudinal study in Japan

**DOI:** 10.3389/fpubh.2024.1454978

**Published:** 2024-09-10

**Authors:** Hiroko Okada, Tsuyoshi Okuhara, Takahiro Kiuchi

**Affiliations:** Department of Health Communication, School of Public Health, The University of Tokyo, Tokyo, Japan

**Keywords:** COVID-19, pandemic, preventive behavior, media information, risk communication

## Abstract

**Objective:**

We aimed to examine the direct effects of exposure to media information about infection-preventing behavior and its indirect effects via interpersonal communication at two time points during the pandemic.

**Methods:**

In August 2020 and August 2021, a web-based survey of Japanese people under a declared state of emergency was conducted. We collected sociodemographic data and data on seven types of exposure to media information, three types of exposure to interpersonal communication, and six types of infection-preventing behavior.

**Results:**

A total of 784 participants completed both surveys. Exposure to information in the mass media decreased over the year, while interpersonal communication about COVID-19-related topics increased. The direct effect of exposure to information in the media about preventive behaviors was statistically significant in the pandemic’s early stages, but this was no longer true after 1 year. The indirect effect via interpersonal communication was statistically significant at both time points.

**Conclusion:**

Our results suggest that the influence of media information on infection-preventing behavior during the pandemic was maintained over time as an indirect effect via interpersonal communication. For risk communication media strategies during pandemics, adopting strategies to generate interpersonal communication will have a sustained effect on preventive behavior.

## 1 Introduction

Due to its prolonged duration, the COVID-19 pandemic seriously affected human health and society (1). In the absence of a cure, the only way to protect oneself was to combine vaccination, which has a limited duration of efficacy, with preventive behaviors such as wearing masks and social distancing (2, 3). As time went on, people experienced “pandemic fatigue” and became less willing to engage in infection-preventing behaviors (4–6). During a pandemic, such decreased individual engagement in preventive behaviors poses a serious threat to public health.

The media is a major source of information in a pandemic because of its access to expert information and ability to disseminate large amounts of information quickly and widely (7–9). In the pandemic’s early stages, many Japanese people paid attention to TV news, web-based news sites, and tabloid TV shows as sources of information (10, 11). When the media communicates effectively and informs the public about facts, people can form accurate risk assessments (12–14). A study conducted during the H1N1 pandemic suggested that attention to the news influences individuals’ likelihood of engaging in preventive behaviors (15). However, some studies support the limited effects theory of the media, which suggests that the direct effects of exposure to media information on individual behavior are limited (16–18). A meta-analysis examining the effects of media campaigns on individual health behaviors suggested that the effects are short term and minor, and that it is unfeasible to try to use media campaigns to change population behavior by as much as 20% (17).

Research on media communication suggests that interpersonal communication mediates the effects of media information on individual behavior (19, 20). For example, the two-step flow model describes a combination of one-to-many communication (e.g., mass media) and one-to-one communication (e.g., interpersonal communication) as changing an individual’s behavior. These mediation models are based on interpersonal communication’s functions for media information of relaying media information to others (social diffusion), providing opportunities to discuss media content in detail, and reinforcing social norms (21). In previous studies on skin cancer and HIV prevention, interpersonal communication was found to mediate the direct effects of exposure to media information on individuals’ perceptions of risk and attitudes toward preventive behavior (17). Another study of a smoking cessation campaign showed that interpersonal discussion after exposure to the media campaign mediated the direct effect of exposure to the media campaign (19). Importantly, previous studies have suggested that the mediated effects of interpersonal communication are greater than the direct effects of exposure to media information in promoting health behaviors. For example, a study during the H1N1 influenza pandemic reported that the mediated effect of personal discussions with family and friends on vaccination intention was greater than the direct effect of exposure to mass media messaging about vaccination (22). Another study of Hong Kong residents during the MERS outbreak reported no direct effect of mass media messaging on preventive behavior and detected only fully mediated indirect effects of interpersonal communication (23). A meta-analysis of media health campaigns found that the odds of achieving health goals were significantly better when there was some interpersonal communication compared to when there was only media campaign exposure (17, 21). Additionally, interpersonal sources of information influence behavioral change relatively quickly, whereas the mass media influences the perceptual and attitudinal aspects of responses more gradually (21).

Therefore, previous studies have suggested that effectively combining information spread by the media and interpersonal communication is the key to encouraging people to engage in necessary health behaviors during prolonged health crises. However, no longitudinal study has examined the long-term effect of exposure to media information on adopting infection-prevention behavior during a prolonged pandemic or looked at how interpersonal communication mediates that effect. This study seeks to examine the direct effects of exposure to media information about COVID-19 prevention behavior and the mediating effects of interpersonal communication regarding such behavior during the COVID-19 pandemic in Japan longitudinally over the one-year period from the time the pandemic was declared. Based on the results, we discuss strategies for increasing the effectiveness of media communication on infection-prevention behaviors by combining them with interpersonal communication.

## 2 Methods

### 2.1 Study design

A longitudinal study was conducted in Japan using a web-based survey administered at two time points: immediately after the declaration of the COVID-19 pandemic in August 2020 and 1 year later in August 2021. The state of emergency declared by the Japanese government was in effect at both times.

### 2.2 Data collection

Based on the platform of Rakuten Insight, Inc., the sample was drawn from a sample of 2.2 million Japanese residents. We used non-probabilistic quota sampling to ensure that the sample was representative of the whole population of Japan. The sample had the same proportions for gender, age, and prefecture as the general Japanese population. Those eligible to participate in the study were (1) people living in areas where a state of emergency had been declared, (2) not infected with COVID-19 themselves and with no family members who were infected, and (3) 18 years of age or older.

We conducted the first survey on 15–16 August 2020 and the second on 15–17 August 2021. Members of the survey panel who met the inclusion criteria were invited via email to respond to the screening questions. As part of the screening process, the participants were required to read a web page outlining our research. Participants were randomly sampled from among those who responded to the screening questions with the same distribution of age group, gender, and place of residence as the general population in Japan. After agreeing to participate, the participants completed an online questionnaire. Before the survey, a pre-survey was conducted on 10 people recruited through snowball sampling to verify the face validity of the questionnaire. Interviews were conducted with the participants of the pre-survey regarding the ease of understanding the questions, the invasiveness of the questions, and the lack of options, and some of the wording of the questionnaire was revised. After the questions were fixed, the first survey was completed by 1,000 participants from eight prefectures that were then in a state of emergency. The second survey was conducted with participants recruited from the first survey. The areas surveyed were under a state of emergency at both times. The data, separated from personal information by the research firm, was provided to the researchers for analysis. An analysis was conducted on the 784 participants who completed both surveys. The follow-up rate was 78.4%.

### 2.3 Measurements

Sociodemographic data (e.g., gender, age, level of education, household income, place of residence, employment status, history of chronic disease, and co-residence) were collected in the first survey. As part of the first survey, health literacy was also assessed. This is because health literacy is associated with seeking information about health and with preventive behavior against infection. We analyzed health literacy using a five-item version of Ishikawa et al.’s (24) Communicative, Critical Health Literacy.

#### 2.3.1 Exposure to media information about COVID-19

The first and second surveys assessed the frequency of exposure to six types of media information channels: newspapers, news websites, non-news websites, social media, TV news, and tabloid TV shows. We asked about the level of attention to COVID-19-related information on each channel in the past week, using a single question adopted from previous studies (e.g., “How much attention did you pay to information about COVID-19 in newspapers?”) (25). Based on a 10-point scale, we rated each item from 1 (paid no attention at all) to 10 (paid a great deal of attention). Cronbach’s alpha for the combined scores was T1 = 0.77, T2 = 0.74.

#### 2.3.2 Interpersonal communication about COVID-19

The frequency of interpersonal communication about COVID-19 with family, friends, and physicians was assessed in the first and second surveys. We asked each subject how often they exchanged COVID-19-related information with each type of person over the past week (e.g., “How often did you exchange information about COVID-19 with your family?”). Based on a 10-point scale, we rated each item from 1 (not at all) to 10 (very often). This question was adopted from previous studies (25). Cronbach’s alpha for the combined scores was T1 = 0.67, T2 = 0.69.

#### 2.3.3 COVID-19 preventive behaviors

The Japanese government recommended preventive behaviors for COVID-19 similar to those endorsed by the World Health Organization (26). Participants in the first and second surveys were asked whether they had engaged in six preventive behaviors recommended by the Japanese government (social distancing, avoiding closed spaces, utilizing ventilation, wearing masks, using hand sanitizer, and washing their hands with soap) (26). Specifically, through a single question, the participants were asked how often they engaged in each of the six COVID-19 prevention behaviors over the past 7 days (e.g., “How often did you wear a mask to prevent COVID-19 over the past 7 days?”). Based on a previous study, items were scored on a 5-point scale ranging from 1 (never) t to 5 (always) (27). For the scaled overall scores, Cronbach’s alpha was T1 = 0.88, T2 = 0.86.

### 2.4 Ethical considerations

A protocol for this study was approved by the Ethics Review Committee at the Graduate School of Medicine, The University of Tokyo (number 11270). A web-based informed consent form was completed by all participants in accordance with the Helsinki Declaration.

### 2.5 Statistical analysis

Descriptive statistics were computed for each sociodemographic variable. Cronbach’s alpha was calculated for all measures. A *t*-test was conducted to examine the changes between the time points for each type of media information exposure and interpersonal communication.

We examined the direct effects of media exposure on prevention behavior and the indirect effects via interpersonal communication using mediation analysis. A mediating analysis is a method for examining the effect of an explanatory variable (*X*) on a outcome variable (*Y*) via a mediating variable (*M*). In this study, mediation analysis was conducted using the procedure proposed by Hayes (28) and the PROCESS macro in SPSS (29). Ordinary least squares path analysis was used to estimate the coefficients of the direct and indirect effects of media exposure on COVID-19 preventive behavior mediated via interpersonal communication. In estimating the coefficients, we adjusted for variables that have been shown in previous studies to influence infection prevention behavior. These covariates included in the analysis were age, gender, level of education, household income, area of residence, employment status, co-residence with family, chronic disease status, and health literacy at Time 1 (T1). We found no interaction between exposure to media information and interpersonal communication on preventive behaviors. The bias-corrected bootstrap method (5,000 random samples) was used to calculate the 95% confidence intervals for the test of indirect effects. This analysis was conducted first cross-sectionally using data from time points T1 and T2, respectively, and then longitudinally using data from T1 for exposure to media information and interpersonal communication and T2 for preventive behavior.

Because of the online survey’s specifications, there were no missing values. Our analysis excluded participants who only responded to the first survey. We conducted all tests on a two-sided basis with a 5% significance level. The data were analyzed using IBM SPSS version 25 (IBM Corp., Armonk, NY, United States).

## 3 Results

### 3.1 Participant characteristics

The participants’ characteristics are presented in Table 1. As stated in the method section, their gender, age, and prefecture of residence were similar to those of the general Japanese population. The proportions of participants with a level of education lower than a university degree and those with a university degree or higher were similar. Approximately half of the participants were full-time employees. Those who were included in the analysis and those who were excluded because they did not take the second survey did not differ statistically significantly in their demographic characteristics.

**Table 1:** Sociodemographic characteristics of participants (*n* = 784).

	*n*	%
Age (Mean, SD)	47.14	12.8
Gender		
Female	388	49.5
Male	396	50.5
Educational level		
Less than high school	12	1.5
High school graduate	156	19.9
Vocational school/Junior collage	175	22.3
Bachelor’s degree	379	48.3
Post baccalaureate degree	62	7.9
Income (US $)		
Less than $15,000	64	8.2
$15,000 to <$43,500	320	49.0
$43,500 or more	334	42.6
Unclear	66	8.4
Employment status		
Employed full-time	415	52.9
Employed part-time	179	22.8
Retired/unemployed	190	24.2
Chronic disease	149	19
Co-residence with family	613	78.2
Health Literacy^†^ (Mean, SD)	3.822	0.6

SD, standard deviation. ^†^Health literacy was measured by the CCHL.

### 3.2 Exposure to media information and interpersonal communication about COVID-19

Table 2 shows the changes in exposure to media information and frequency of interpersonal communication over the year. Exposure to media information tended to decrease over the year. The types of media information to which the participants had the highest degree of exposure were news information obtained from TV and websites for both T1 and T2. Exposure to old media (TV and newspapers) showed a statistically significant decrease over the year (*p* < 0.001 respectively). On the other hand, exposure to new media such as social media and video sharing sites showed a statistically significant increase over the year (*p* = 0.02, *p* < 0.001 respectively). There was a statistically significant increase in the frequency of interpersonal communication about COVID-19-related topics among all subjects with their physicians, family, and friends over the year (*p* < 0.001 respectively).

**Table 2:** Changes in exposure to each type of COVID-19 media information and interpersonal communication (*n* = 784).

	T1		T2		Differences				
	Mean	SD	Mean	SD	Mean	SD	95%CI		p^*^
Media information									
Newspaper	5.25	3.373	4.82	3.329	−0.432	2.825	−0.630	−0.234	<0.001
TV news	7.33	2.898	7.00	2.801	−0.325	2.379	−0.492	−0.158	<0.001
TV tabloid shows	6.02	2.996	5.67	2.910	−0.352	2.458	−0.524	−0.180	<0.001
News websites	6.78	2.609	6.68	2.528	−0.092	2.606	−0.275	0.091	0.324
Government and medical professional official websites	5.61	3.154	4.79	2.857	−0.813	3.371	−1.049	−0.576	<0.001
Video sharing sites	3.52	2.700	4.16	3.025	0.649	3.022	0.437	0.861	<0.001
Social media	3.68	2.784	3.92	2.962	0.239	2.874	0.037	0.440	0.020
Interpersonal communication									
Physicians	3.18	2.527	4.01	3.020	0.825	3.073	0.610	1.041	<0.001
Family	6.67	2.452	7.24	2.648	0.570	2.309	0.408	0.732	<0.001
Friends	5.40	2.493	5.91	2.638	0.506	2.502	0.331	0.682	<0.001

*Paired *t*-test.

**Figure 1:**
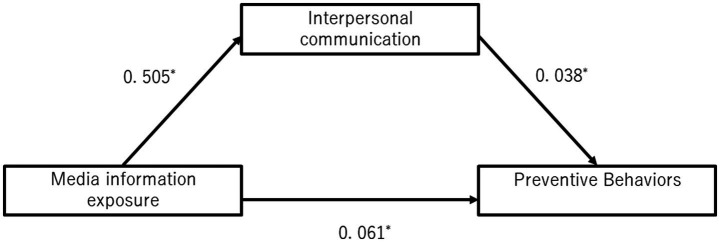
The direct effect of exposure to media information on prevention behavior and indirect effect mediated by interpersonal communication at the first survey. *p < 0.05 adjusted for age, gender, education, income, area of residence, employment status, co-residence with family, chronic disease status, and health literacy by multiple regression analysis.

Figure 1 shows the results of the mediation analysis in which the outcome variable was preventive behavior, the explanatory variable was exposure to media information, and interpersonal communication was the mediating variable in the first survey. There was a significant direct effect of media information exposure on preventive behavior (0.061, 95% CI = 0.025–0.096). Media information exposure was also shown to be significantly associated with preventive behavior indirectly through interpersonal communication (0.038, 95% CI = 0.017–0.06). The effect of media information exposure on the interpersonal communication was also significant (0.505, 95% CI = 0.437–0.572).

**Figure 2:**
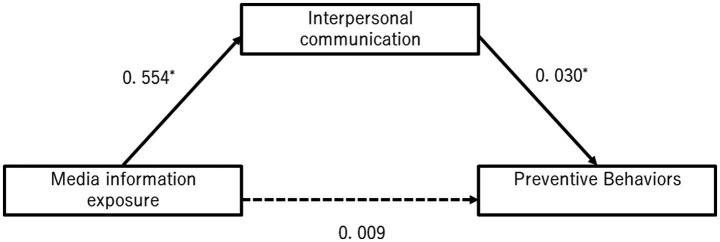
The direct effect of exposure to media information on prevention behavior and indirect effect mediated by interpersonal communication at the second survey. *p < 0.05 adjusted for age, gender, education, income, area of residence, employment status, co-residence with family, chronic disease status, and health literacy by multiple regression analysis.

Figure 2 shows the results of the mediation analysis in which the outcome variable was preventive behavior, the explanatory variable was exposure to media information, and interpersonal communication was the mediating variable in the second survey. There was no significant direct effect of media information exposure on preventive behavior (0.009, 95% CI = −0.026–0.045). On the other hand, media information exposure was found to be significantly associated with preventive behavior, indirectly through interpersonal communication (0.03, 95% CI = 0.009–0.052). The effect of media information exposure on the interpersonal communication was also significant (0.554, 95% CI = 0. 476–0.632).

## 4 Discussion and conclusion

### 4.1 Discussion

This study examined the direct effects of exposure to media information about COVID-19 on engagement in infection-prevention behavior and the indirect effects mediated through interpersonal communication at two time points among residents of Japan under the COVID-19 state of emergency. Understanding longitudinal changes in the effects of people’s exposure to information on their infection-prevention behavior will enhance communication strategies to maintain and promote public health compliance during prolonged pandemics.

Regarding exposure to media information about COVID-19 during the first year of the pandemic, there was a high level of exposure, particularly to news information on TV and on the web. This result supported previous studies indicating that people consider the news an important source of information during disease outbreaks (9, 15). During the following year, exposure to information about COVID-19 in the mass media decreased. This may be partly due to a decrease in the frequency of media coverage over time. On the other hand, interpersonal communication on topics related to COVID-19 increased over the year for all targets. This suggests that in a pandemic lasting longer than a year, people’s information-seeking behavior may change between the start of the pandemic and 1 year later. One previous qualitative study reported that over the course of the pandemic, the content of people’s interpersonal communication shifted from content based on media reports to content based on personal experience (30). In a pandemic’s early stages, people communicate about the unknown disease and base their communication on proxy experiences through the media. However, as the number of infected people increases, these proxy experiences are likely to be replaced, or at least supplemented, by direct experiences. In other words, when people do not have access to the experiences of those close to them, they will obtain more information from the media, but when they have access to the experiences of those close to them, they may seek more exposure to personal information. This may explain why exposure to media information about COVID-19 decreased over the year, while exposure to interpersonal communication and new media that allow people to share personal information about COVID-19 increased.

Regarding the direct effect of exposure to information in the media on preventive behavior, a significant effect was detected at T1 but not at T2. Based on cultivation theory, which states that the media cultivates social reality, norms, and values, frequent media users interpret the presumed frequency, likelihood, and characteristics of events described in the media as reality, thus influencing norms (e.g., descriptive norms). Moreover, previous studies have reported that risk emotions such as fear and anxiety motivate information-seeking and protective behaviors (31, 32). For example, a previous study on H1N1 reported that media reports evoking fear tended to draw increased public attention to the media (15). COVID-19 spread rapidly, and the numbers of deaths and severe cases were reported daily in the media (9). In the early stages of the pandemic, the high exposure of many people to such information may have led to fear and a high estimate of their own likelihood of infection, which may have led to increased monitoring of media information and protective action (33). However, contrary to these predictions, the present study showed that even during the pandemic, the direct effect of the media decreased over time. The following reasons may explain why the direct effect of the media on public preventive behavior was not sustained for long. First, during the pandemic, the public was exposed to a high frequency of messages promoting preventive behavior disseminated through the media. However, it is known that a higher frequency of exposure to persuasive messages does not necessarily produce good persuasive effects (34). When individuals are exposed to similar messages beyond the desired frequency, the result can be message fatigue (35). Studies on issues such as smoking cessation, dental floss use, and disease prevention programs have reported that such fatigue can lead to non-compliance with recommended behavior through both active resistance (reactance) and passive resistance (neglect) (36–38). A study conducted during the COVID-19 pandemic indicated that people exposed to a high volume of messages may have developed message fatigue, leading them to disregard this information and reduce their infection-prevention behaviors (39, 40). Second, media reports of the rapidly increasing number of infections and deaths unintentionally appealed to the public’s fear of this infectious disease. In the early stages of the pandemic, this may have encouraged public preventive behavior, but studies of emotions and coping strategies suggest that people adapt to these emotions through defiance mechanisms such as reframing and denial (41). This study’s results may reflect the characteristics of a prolonged pandemic in which the direct effect of media information on public preventive behavior was reduced by continuing to monotonously repeat data reporting and persuasive messages.

However, regarding the indirect effect of exposure to media information on prevention behavior mediated by interpersonal communication, statistically significant effects were found for both T1 and T2. Combined with the result that the direct effect of media information exposure detected in T1 was not detected in T2, this suggests that the effect of exposure to information in the media on preventive behavior shifted from a direct effect to an indirect effect through fully mediated interpersonal communication during the year of the pandemic. Based on the two-step flow model, media messages are spread by opinion leaders who are more exposed to the media; through their relationships, these opinion leaders spread the message to groups that were not exposed to the original message (42). In the early stages of the pandemic, most people were actively exposed to media information, including those who do not actively collect health information in normal times. In a situation in which the public was faced with a crisis, the number of people directly exposed to media information increased, which may have resulted in a situation in which the one-step flow (direct influence by media information) and the two-step flow (influence via interpersonal communication) coexisted. Over time, the information-seeking behavior of non-opinion leaders may have calmed down, resulting in the media information flow shifting to a complete two-step flow. In other words, media information has the potential to continue to influence preventive behavior over time; even if its direct effect diminishes, if it can be mediated through interpersonal communication. Previous studies of health campaigns have reported that the effect on individual behavior was greater when interpersonal communication was generated from the campaign in addition to direct exposure to information from the campaign (21). To sustain the long-term effects of media information on individual preventive behavior, including content that triggers interpersonal communication may be an effective approach.

Previous studies have suggested that several public health communication strategies are effective in generating interpersonal communication through the media. The first is to provide explicit discussion prompts that encourage discussion with individuals close to the recipient of the message. For example, the National Youth Anti-Drug Media Campaign encouraged parents to talk with their children in the expectation that increased parent–child communication about drugs would stop drug use (43). This triggered more frequent parent–child conversations. Presenting messages that explicitly include the target and content of the conversation to be generated has the potential to increase conversations about media information. Second, many studies have shown that emotionally stimulating content stimulates interpersonal communication (44). People often talk with others about emotional events (45). Emotion has been reported to trigger evaluation and diffusion of health topics in campaigns (46). Topics with the ability to evoke emotions have the potential to create public buzz and communication opportunities. Third, the use of narratives in public health messages may also be effective. Previous studies examining the effectiveness of public health messages in social media have reported that narrative messages directly influence intentions to share in interpersonal communication (47). At the same time, it has been reported that narrative messages are less likely to generate psychological reactance because it is difficult to perceive the sender’s persuasive intentions. Narratives may be more easily accepted and spread more readily under pandemics, where psychological reactance to persuasive messages is more likely to develop.

In summary, the results of this study suggest that the influence of media exposure on preventive behavior during a pandemic may shift from a direct effect in the early stages to an indirect effect mediated by interpersonal communication as the pandemic becomes more prolonged. To promote infection-prevention behavior for a prolonged duration using the media, including content that leads to the generation of interpersonal communication may be an effective strategy.

Several limitations should be considered when interpreting this study’s results. First, because this is an observational study, a causal relationship between exposure to media information and interpersonal communication and infection-prevention behavior cannot be established based on its results. Furthermore, because non-probability sampling was adopted, there are limitations to the representativeness of the sample. Therefore, we relied on previous studies and existing theories in our discussion. However, considering that it is quite difficult to conduct a randomized controlled trial in a crisis situation, the results of this study represent important findings. Second, the participants all had to have access to the internet survey form to complete the survey, and their level of education was likely to be higher than that of the general Japanese public. Additionally, as the data was collected through questionnaires, there is a possibility of self-reporting bias. Finally, single-item, unvalidated scales were used in this study for exposure to media information, frequency of interpersonal communication, and engagement in preventive behaviors. We adopted items commonly used in previous studies to enable comparisons with the results of other infectious disease pandemics and with results from other countries. In the future, it is expected that scales measuring these factors will be developed. Despite these limitations, this is the first study to report changes between two time points during the pandemic in the direct effects of information in the media on COVID-19 prevention behavior and the indirect effects mediated by interpersonal communication. The conflict between humanity and emerging infectious diseases will continue in the future. And in the case of a future pandemic, new information media will become more widespread, and the infodemic may become an even more serious problem. This study will provide a perspective and the importance of utilizing interpersonal communication, which is the basis of communication, in public health communication strategies in the event of a future pandemic.

### 4.2 Conclusion

In the early stages of a pandemic, the media had a direct effect on people’s preventive behaviors. However, when a pandemic becomes prolonged, the effect is maintained only as an indirect effect, fully mediated by interpersonal communication.

Mass media is one of the most useful tools for rapidly providing information to large numbers of people. As observed during the COVID-19 pandemic, risk communication via the mass media is a major channel for governments and other risk communicators. The results of exposure to the media for the participants in this study also suggest that the general public attempted to access information about COVID-19 through the mass media in the early stages of the pandemic. The media is an important channel for risk communicators to promote infection-prevention behavior during a pandemic. This study suggests a possible strategy for sustaining the effectiveness of risk communication through the media over a long period of time during a pandemic. This means generating interpersonal communication among the public on health topics through media information. To sustain the effectiveness of risk communication through the media, it may be useful to incorporate triggers that generate interpersonal communication, such as explicit prompts to communicate with people close to the message recipients, including emotionally evocative content, and incorporating narrative messages. Risk communicators have been able to take advantage of the media’s ability to communicate immediately with huge numbers of people during pandemics to rapidly disseminate accurate information. In addition, if the “emotionality” of the media can be leveraged, risk communications will continue to influence the behavior of greater numbers of people for longer periods.

## Data Availability

The raw data supporting the conclusions of this article will be made available by the authors, without undue reservation.
